# LDL regulates intestinal stem cell homeostasis via PPAR pathway

**DOI:** 10.1016/j.jlr.2025.100826

**Published:** 2025-05-14

**Authors:** Ruicheng Shi, Wei Lu, Zhiming Zhao, Bo Wang

**Affiliations:** 1 Department of Comparative Biosciences, College of Veterinary Medicine, University of Illinois at Urbana-Champaign, Urbana, IL, USA; 2 Division of Nutritional Sciences, College of Agricultural, Consumer and Environmental Sciences, University of Illinois at Urbana-Champaign, Urbana, IL, USA; 3 Cancer Center at Illinois, University of Illinois at Urbana-Champaign, Urbana, IL, USA

**Keywords:** fatty acid oxidation, hyperlipidemia, ISCs, LDL, PPAR

## Abstract

Epidemiological studies have highlighted a strong association between hyperlipidemia and an increased risk of cancer in the gut. Intestinal stem cells (ISCs) have been demonstrated as the cells of origin for tumorigenesis in the gut. However, the impact of hyperlipidemia on ISC homeostasis remains unclear. Here, we show that hyperlipidemia induced by LDL receptor (*Ldlr*) deficiency enhances ISC proliferation in vivo. Additionally, LDL treatment impairs organoid survival but increases ISC stemness ex vivo, as evidenced by the formation of poorly differentiated spheroid and higher ISC self-renewal capacity. Mechanistically, LDL treatment activates PPAR pathways, and pharmacological inhibition of PPAR and its downstream targets, including CPT1A and PDK4, mitigates the effect of LDL on ISCs. These findings demonstrate that hyperlipidemia modulates ISC homeostasis, providing new insights into the mechanism linking hyperlipidemia with tumorigenesis in the gut.

## Graphical abstract

**Figure:**
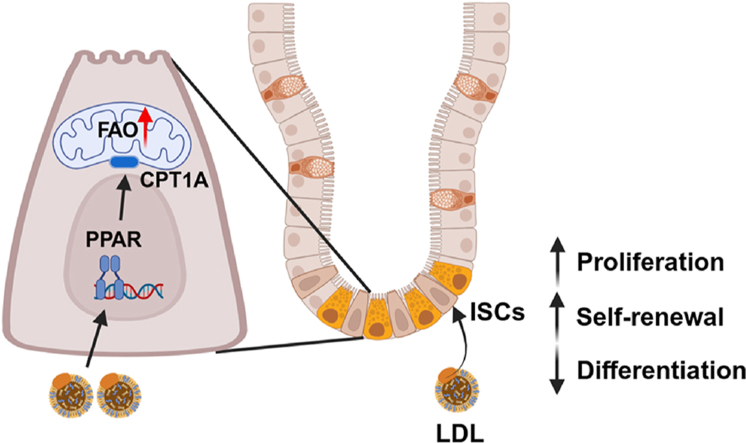

Epidemiological studies have revealed strong correlation between dietary lipid intake and hyperlipidemia and cancer risk in the gut (1, 2, 3, 4, 5, 6). A higher incidence of colorectal neoplasms has been observed in patients with coronary artery disease (7), a condition often associated with hyperlipidemia, particularly elevated LDL cholesterol levels, which have also been identified as a significant risk factor for the recurrence of colorectal cancer (CRC) in human patients (8). Furthermore, several genes involved in cholesterol de novo biosynthesis pathway, regulated by the transcription factor SREBP2, have been shown to be upregulated in CRC tumors and associated with poor prognosis in human patients (9, 10). Taken together, these observations strongly indicate a potential role of lipid metabolism in the initiation and progression of CRC.

Intestinal stem cells (ISCs) have been demonstrated to be the cells of origin for most precancerous adenomatous lesions in the gut (11, 12). Studies utilizing mouse models and human ex vivo organoid culture have demonstrated that mutations in ISCs are sufficient to initiate the tumorigenesis from these cells (11, 13). In physiology, ISCs regularly divide to produce highly proliferative progenitor cells known as transit-amplifying cells, which further divide four to five times and differentiate into intestinal epithelial cells (14), including absorptive enterocytes and secretory goblet cells, enteroendocrine cells, and Paneth cells. Proper balance between ISC self-renewal and differentiation is crucial to preserve the integrity of the intestinal epithelium and maintain its homeostasis, whose dysregulation has been shown to contribute to the development of various intestinal pathologies, such as inflammatory bowel disease and gastrointestinal cancers. Research has demonstrated that compromised epithelial integrity in the gut exposes ISCs to environmental mutagens, thereby promoting tumorigenesis (15). Conversely, the dynamics and functionality of ISCs greatly influence cancer initiation (16).

The homeostasis of ISCs is intricately regulated by signals from the surrounding microenvironment, including niche factors provided by Paneth cells and subepithelial mesenchymal cells (17). Recent studies have highlighted the impact of nutritional factors, such as calorie restriction, high-fat diet, fatty acid β-oxidation (FAO), and ketone bodies on ISC self-renewal and tumorigenesis (18, 19, 20, 21), underscoring the interconnection between nutritional status and metabolism with ISC function. Our previous studies have demonstrated that elevating cellular cholesterol levels through dietary cholesterol intake or enhancing endogenous cholesterol biosynthesis via SREBP-2 overexpression promotes ISC self-renewal and proliferation, as well as *Apc*^*Min/+*^-induced tumorigenesis in the intestine (22). In contrast, blocking cholesterol production significantly hinders crypt development. Deletion of SREBP cleavage-activating protein in the mouse intestines prevents SREBP activation and lipid production, leading to severe crypt development failure and enteropathy (23). These findings demonstrate the profound impacts of cellular cholesterol metabolism on ISC homeostasis. However, whether and how circulating lipids impact ISC function have been poorly investigated.

In this study, we investigated the impact of circulating LDL on ISC proliferation. Deletion of LDL receptor (*Ldlr*), a key receptor for plasma LDL clearance, in mice elevates ISC activities in vivo. Furthermore, we demonstrated ex vivo that LDL impairs initial organoid formation but ultimately enhances stemness and self-renewal capabilities. Taken together, these findings highlight the complex role of LDL on ISC homeostasis and self-renewal.

## Materials and Methods

### Animal models

*Ldlr*^*−/−*^ and C57BL/6 mice were purchased from the Jackson Laboratory (Bar Harbor, ME). All animal procedures were conducted in compliance with protocol (#24157) approved by the IACUC at the University of Illinois at Urbana—Champaign. All mice were housed under pathogen-free conditions in a temperature-controlled room with a 12 h light/dark cycle. All mice were fed on a chow diet. During harvest, different regions (duodenum, jejunum, and ileum at a length ratio of 1:2:1) from small intestine samples were collected and immediately flushed with cold PBS with a syringe. All samples were snap-frozen in liquid nitrogen and stored at −80°C, fixed in 10% formalin, or frozen in OCT for cryosectioning. Plasma was collected by retro-orbital bleeding followed by centrifugation. Plasma lipids were measured with Wako Free Cholesterol E kit and Wako Cholesterol E kit (FUJIFILM, Richmond, VA). Crypt lipids were extracted with modified Bligh-Dyer lipid extraction, and free cholesterol content was measured with Amplex Red Cholesterol Assay Kit (Thermo Fisher Scientific, Waltham, MA). Tissue histology was performed in the University of Illinois at Urbana—Champaign Comparative Biosciences Histology Laboratory.

### Crypt isolation

Briefly, mice were sacrificed, and duodenum and jejunum were immediately removed, flushed with cold PBS, and cut open longitudinally. The tissues were rinsed twice with cold PBS and gently rocked with PBS containing 2.5 mM EDTA for 30 min at 4°C. Intestines were then vortexed vigorously for 30 s in 3 s pulses to release crypts. The mixture was then sat on ice for 10 min to allow the villi to settle down. Crypts in the supernatant were passed through a 70 μm strainer (BD Biosciences, Bedford, MA) and centrifuged at 100 *g* for 3 min to pellet the crypts. The pelleted crypts were then used for organoid culture or other experiments.

### 3D organoid culture

The collected pellet was resuspended in advanced DMEM/Ham’s F12 (Thermo Fisher Scientific, Waltham, MA). Crypts were quantified under a microscope and embedded in Matrigel (Thermo Fisher Scientific, Waltham, MA) at a concentration of 10–15 crypts/μl. Around 150 crypts were seeded in each well in a dome-shaped matrix on a 48-well plate and cultured in IntestiCult Organoid Growth Medium (StemCell Technology, Vancouver, BC). The growth medium was changed every other day.

### Organoid measurement

The growth of organoids was evaluated by quantifying total organoid numbers per well under the microscope 5 days postseeding. Spheroids were distinguished from other budding organoids and quantified under light microscopy.

### Secondary organoid assay

The primary organoids were washed in PBS once and mechanically disrupted by going through a syringe with a 21 G needle 30 times. The cells were then centrifuged, resuspended in Matrigel, and reseeded as described above. Secondary organoids were quantified at day 3.

### Organoid treatment

Organoids were treated with the following chemicals: LDL (25, 50 μg/ml; Lee Biosolutions, Maryland Heights, MO), etomoxir (100 μM; Cayman Chemical Company, Ann Arbor, MI), GSK3787 (2 μM; MedChemExpress, Monmouth Junction, NJ), and dichloroacetate (DCA) (20 mM, Thermo Fisher Scientific, Waltham, MA). All chemicals were added 24 h postseeding except for LDL.

### RNA-sequencing analysis

RNA-sequencing (RNA-Seq) analysis was performed in organoids treated with PBS or LDL (50 μg/ml) for 5 days. In brief, organoids were washed once with PBS, and total RNAs were isolated using TRIzol reagent (Invitrogen, Waltham, MA). Ribosomal RNA was removed with the Ribozero kit (Illumina, San Diego, CA). The RNA-Seq libraries were prepared with TruSeq Stranded mRNAseq Sample Prep kit (Illumina). The libraries were pooled; quantitated by quantitative PCR and sequenced on one SP lane for 101 cycles from one end of the fragments on a NovaSeq 6000. Fastq files were generated and demultiplexed with the bcl2fastq v2.20 Conversion Software (Illumina). Fastq files were aligned to mouse reference genome GRCm39 using STAR 2.7.10b. The count matrix was created using featureCounts, and differential expression analysis was performed using DESeq2. For Gene Ontology analysis, genes with adjusted *P* values less than 0.001 were selected for the enrichment analysis. RNA-Seq data have been deposited at Gene Expression Omnibus (accession number: GSE269253).

### Ethynyl-2-deoxyuridine labeling and staining

For ethynyl-2-deoxyuridine (EdU) staining, mice were i.p. injected with EdU (10 mg/kg) 2 h prior to tissue collection. Intestine tissues were embedded in OCT and cryosectioned (10 μm). EdU staining was conducted using Click-iT EdU imaging kit (Thermo Fisher Scientific, Waltham, MA) according to the manufacturer’s protocol. Briefly, slides containing mounted frozen sections were allowed to thaw to room temperature and then fixed with 4% paraformaldehyde in PBS for 15 min. Slides were then washed twice with 3% BSA in PBS and permeabilized with 0.5% Triton X-100 in PBS for 20 min. The slides were washed twice with 3% BSA in PBS and incubated with a Click-iT reaction cocktail (Thermo Fisher Scientific; catalog no.: C10337) containing Click-iT reaction buffer, CuSO_4_, Alexa Fluor® 488 Azide, and reaction buffer additive for 30 min in dark. The sections were washed once more with 3% BSA in PBS and incubated with 5 μg/ml Hoechst 33342 for 10 min. The slides were then washed twice with PBS and mounted with ProLong™ Gold Antifade Mountant (Thermo Fisher Scientific, Waltham, MA).

### Immunohistochemistry and immunofluorescence

All staining was conducted using samples embedded in paraffin. For olfactomedin 4 (OLFM4) staining, the paraffin sections were rehydrated and subjected to antigen retrieval for 20 min in near-boiling citric buffer (pH 6.0). Slides were blocked with 10% goat serum for 1 h at room temperature and incubated with anti-OLFM4 (1:350 dilution; Cell Signaling Technologies), anti-Ki-67 (1:50 dilution; Abcam), anti-β-catenin (1:50 dilution; BD Bioscience), or anti-Lysozyme (1:400 dilution; Dako) overnight at 4°C. Slides were then washed and incubated with biotin-conjugated secondary antibodies (Vector Laboratories, Newark, CA), and signals were developed with an ABC kit (Vector Laboratories, Newark, CA).

### Quantitative PCR

Briefly, small intestine tissue was homogenized with TissueLyser II (Qiagen, Hilden, Germany), and total RNA was extracted with TRIzol reagent. Complementary DNA was synthesized, and gene expression was quantified by CFX384 Touch Real-Time PCR Detection System (Bio-Rad, Hercules, CA) with SYBR Green (Bio-Rad, Hercules, CA). Gene expression levels were normalized to 36B4.

### Statistical analysis

Sample sizes were determined based on previous studies and preliminary results. All results were confirmed in at least two different batches of mice. Results from quantitative experiments were expressed as means ± SEM. GraphPad Prism 10 (GraphPad Software, Inc, San Diego, CA) was used for all statistical analyses. Where appropriate, significance was calculated by paired or unpaired Student's *t*-test and one- or two-way ANOVA with Tukey's or Sidak's multiple comparison test.

## Results

### *Ldlr* deficiency promotes crypt proliferation in vivo

**Fig. 1:**
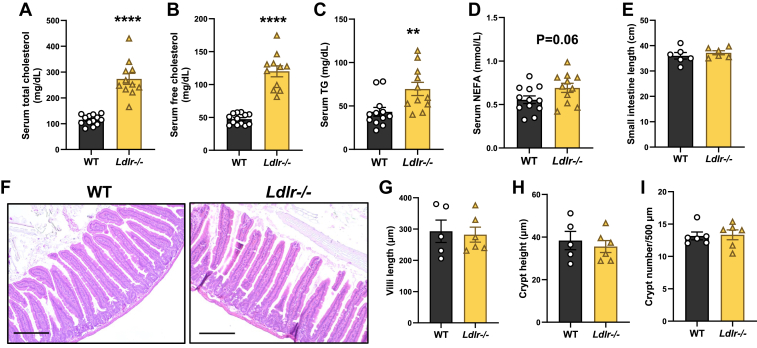
*Ldlr* deficiency-induced hyperlipidemia does not have a dramatic impact on intestinal morphology. A–D: Serum total cholesterol (A), free cholesterol (B), triglyceride (TG) (C), and NEFA (D) levels in WT and *Ldlr*^−/−^ mice (n = 11–13 mice/group). E: Small intestine length of WT and *Ldlr*^*−/−*^ mice (n = 6 mice/group). F: Representative H&E images of WT and *Ldlr*^*−/−*^ mice jejunum. Scale bar represents 100 μm. G–I: Quantification of villi length, crypt height, and crypt density of WT and *Ldlr*^*−/−*^ jejunum (n = 5–6 mice/group). Values are means ± SEM. Statistical analysis was performed with Student's *t*-test. ∗∗*P* < 0.01 and ∗∗∗∗*P* < 0.0001.

To evaluate the impact of hyperlipidemia on ISC homeostasis, we employed an *Ldlr* knockout mouse model (*Ldlr*^*−/−*^), which is known to develop hyperlipidemia, particularly elevated LDL-cholesterol, on a chow diet (24). As expected, lipid measurement showed a marked increase in serum cholesterol and triglyceride levels in *Ldlr*^−/−^ mice compared to *Ldlr*^+/+^ WT mice (Fig. 1A–C). In contrast, serum NEFA levels were only slightly increased in *Ldlr*^−/−^ mice (Fig. 1D). *Ldlr*^−/−^ mice exhibited similar intestine length and comparable intestine morphology, including villi length, crypt height, and crypt density, compared to WT mice (Fig. 1E–I).

**Fig. 2:**
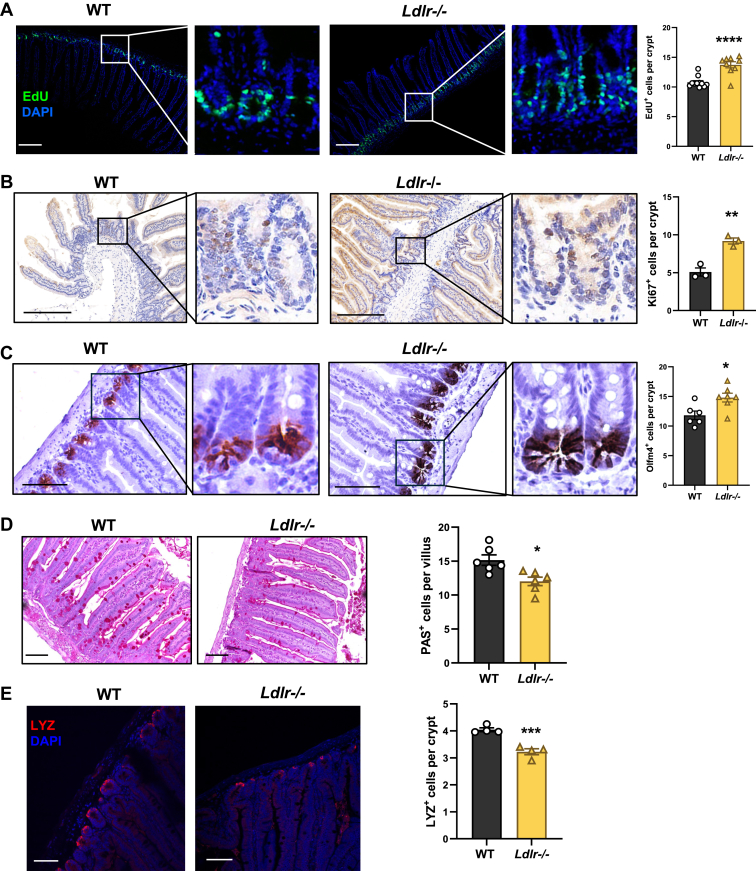
*Ldlr* deficiency enhances crypt proliferation. A: Representative images and quantification of EdU-positive proliferating cells in WT and *Ldlr*^*−/−*^ crypts (n = 9–11 mice/group). Scale bar represents 100 μm. B: Representative images and quantification of Ki67-positive proliferating cells in WT and *Ldlr*^*−/−*^ crypts (n = 3 mice/group). Scale bar represents 100 μm. C: Representative images and quantification of OLFM4-positive stem cells in WT and *Ldlr*^*−/−*^ crypts (n = 6–7 mice/group). Scale bar represents 100 μm. D: Representative images and quantification of periodic acid-Schiff staining of goblet cells in WT and *Ldlr*^*−/−*^ villi (n = 6 mice/group). Scale bar represents 100 μm. E: Representative images of lysozyme (LYZ) immunofluorescence staining and quantification of LYZ-positive Paneth cells in WT and *Ldlr*^*−/−*^ crypts (n = 4 mice/group). Scale bar represents 100 μm. Values are means ± SEM. Statistical analysis was performed with Student's *t*-test. ∗*P* < 0.05, ∗∗∗*P* < 0.001, and ∗∗∗∗*P* < 0.0001.

Interestingly, although *Ldlr*^−/−^ showed no overt phenotype in the intestine, EdU pulse-labeling experiments revealed a ∼30% increase in EdU-positive proliferating cells in the crypts of *Ldlr*^*−/−*^ mice compared to WT (Fig. 2A). Immunohistochemistry analysis of Ki67 revealed ∼1.8-fold increase in Ki67-positive nuclei in the crypts of *Ldlr*^*−/−*^ intestine (Fig. 2B). Furthermore, immunostaining of OLFM4, a robust ISC marker (25), showed a significant increase in OLFM4-positive ISCs in *Ldlr*^−/−^ crypts (Fig. 2C). ISC proliferation and differentiation are closely correlated. We next examined the impact of *Ldlr* deficiency on ISC differentiation. Loss of *Ldlr* resulted in a 33% decrease in periodic acid-Schiff-labeled goblet cells (Fig. 2D) and a 20% reduction in the number of lysozyme-positive Paneth cells (Fig. 2E). These data indicate that *Ldlr* deficiency promotes ISC proliferation but impairs their differentiation toward the secretory cell lineage.

**Fig. 3:**
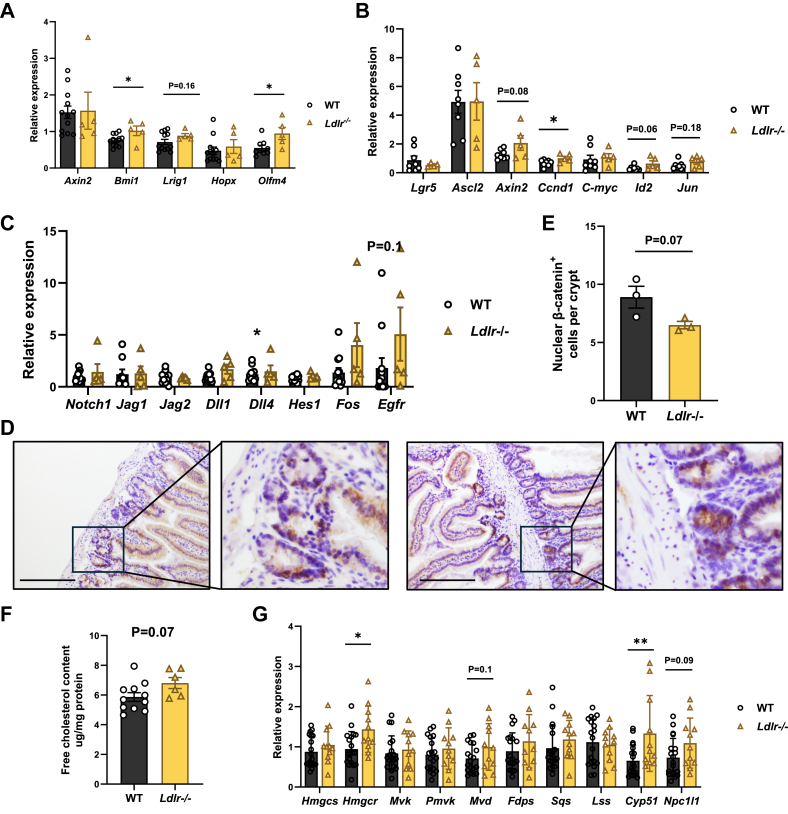
The expression of stem cell markers, genes involved in WNT signaling pathway and cholesterol metabolism in WT and *Ldlr*^*−/−*^ mice. A: Real-time PCR analysis of ISC markers in crypts isolated from WT and *Ldlr*^−/−^ mice (n = 5–12 mice/group). B and C: Real-time PCR analysis of WNT, Notch, and EGF pathway genes in crypts isolated from WT and *Ldlr*^−/−^ mice (n = 5–12 mice/group). D and E: Representative images of β-catenin immunostaining and quantification of nuclear β-catenin in WT and *Ldlr*^−/−^ crypts (n = 3 mice/group). Scale bar represents 100 μm. F: Cellular free cholesterol levels in crypts isolated from WT and *Ldlr*^−/−^ mice (n = 6–11 mice/group). G: Real-time PCR analysis of genes involved in cholesterol biosynthesis in crypts isolated from WT and *Ldlr*^−/−^ mice (n = 11–19 mice/group). Values are means ± SEM. Statistical analysis was performed with Student's *t*-test. ∗*P* < 0.05 and ∗∗*P* < 0.01.

Consistent with the expansion of ISCs in *Ldlr*^−/−^ intestine, real-time PCR analysis of isolated crypts revealed a significant upregulation of *Bmi1* and *Olfm4* in the absence of *Ldlr*, whereas the expression of other ISC markers, including *Axin2*, *Lrig1*, and *Hopx*, remained comparable between *Ldlr*^−/−^ and WT mice (Fig. 3A). The WNT, Notch, and EGF pathways are well established to drive ISC proliferation and differentiation. However, no significant differences were observed between WT and *Ldlr*^*−/−*^ crypts in the expression of several key mediators and downstream targets of these signaling pathways, except for *Ccnd1* and *Dll1* (Fig. 3B, C). Immunostaining of β-catenin revealed a trend of decrease in nuclear β-catenin signals between WT and *Ldlr*^*−/−*^ crypts, indicating that Wnt signaling pathway unlikely contributes to enhanced ISC proliferation in the absence of *Ldlr* (Fig. 3D, E). These data suggest that the altered proliferation and differentiation of ISCs in *Ldlr*^*−/−*^ crypts is unlikely driven by these canonical pathways.

Previous studies have demonstrated that elevated cholesterol synthesis and excess cellular cholesterol levels induce ISC hyperproliferation (22). To test if the increased ISC proliferation in *Ldlr*^*−/−*^ mice may be attributed to elevated cellular cholesterol levels, we measured the free cholesterol content in the crypts. Indeed, compared to WT crypts, *Ldlr*^*−/−*^ crypts exhibited a slight increase (17%) in free cholesterol contents (Fig. 3F). Moreover, real-time PCR analysis revealed a significant upregulation in the expression of several key genes in the SREBP2 pathway, including *Hmgcr* and *Cyp51* (Fig. 3G), likely as a compensatory response to the defect in LDL-cholesterol uptake.

### LDL treatment impairs organoid survival but enhances their self-renewal capacity

**Fig. 4:**
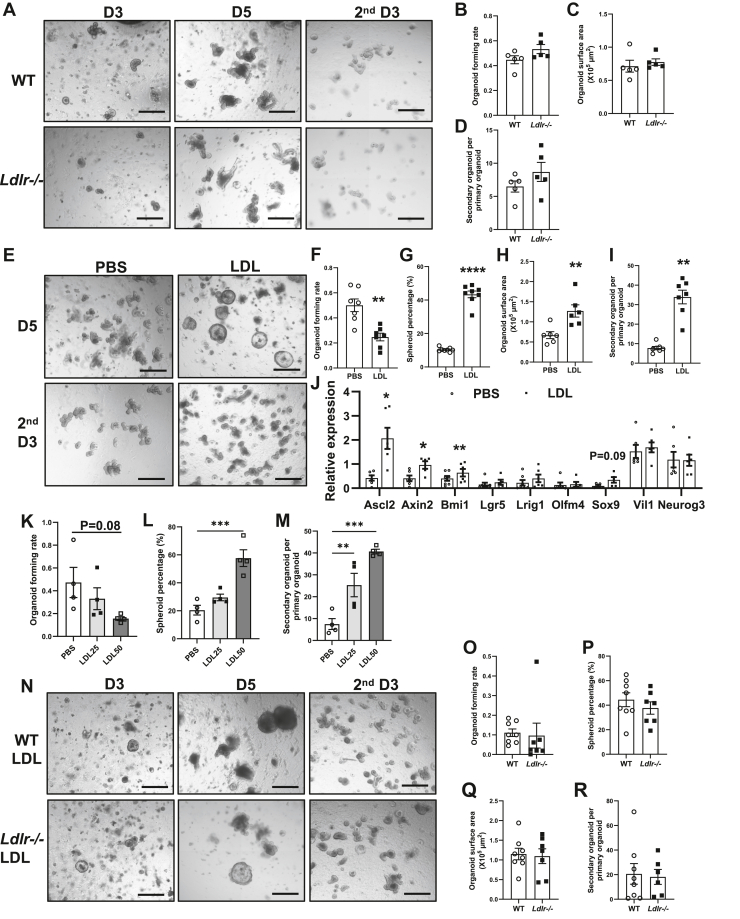
LDL treatment reduces organoid survival but enhances their self-renewal capacity. A: Representative images of organoids derived from crypts isolated from WT and *Ldlr*^*−/−*^ jejunum. B–D: Quantification of organoid-forming rate (B), surface area (C), and secondary organoid number per primary organoid (D) from A. Each dot represents the mean of three to four wells from one mouse (n = 5 mice/group). E: Representative images of organoids derived from crypts isolated from C57BL/6J mice and treated with control (PBS) or LDL (50 μg/ml) for 5 days. F–I: Quantification of organoid-forming rates (F), the percentages of spheroids (G), organoid surface areas (H), and secondary organoid-forming rates (I) from E. Each dot represents the mean of three to four wells from one mouse (n = 6–8 mice). J: Real-time RT-PCR analysis of stemness and lineage marker genes in PBS- and LDL-treated organoids. Each dot represents data from one mouse (n = 6). K–M: Quantification of growth and secondary organoid formation of organoids treated with PBS and different concentrations of LDL treatment. Each dot represents the mean of three to four wells from one mouse (n = 4 mice). N: Representative images of organoids derived from crypts isolated from WT and *Ldlr*^*−/−*^ mice and treated with LDL (50 μg/ml) for 5 days. O–R: Quantification of organoid-forming rates (O), the percentages of spheroids (P), organoid surface areas (Q), and secondary organoid-forming rates (R) from N. Each dot represents the mean of three to four wells from one mouse (n = 7–8 mice). Scale bars in A, E, and N represent 400 μm. Values are means ± SEM. Statistical analysis was performed with paired Student's *t*-test. ∗∗*P* < 0.01 and ∗∗∗∗*P* < 0.0001.

To further investigate the effect of *Ldlr* deficiency on ISC proliferation, we performed ex vivo culture to evaluate the ability of isolated crypts to form organoids in 3D culture as described previously (26). Surprisingly, loss of *Ldlr* did not affect organoid-forming rate, organoid size, or self-renewal ability, as assessed by the secondary organoid-forming rate per primary organoid (Fig. 4A–D). These findings suggest that the impact of *Ldlr* deficiency on ISC proliferation in vivo is likely not intrinsic to the ISCs themselves but may instead be a secondary effect of hyperlipidemia.

*Ldlr* deficiency is known to elevate lipid levels in ApoB-rich LDL lipoproteins. We wondered whether hyperlipidemia may directly enhance ISC function. To test if lipoproteins directly affect ISC proliferation and self-renewal, we cultured crypts isolated from WT mice in the presence of LDL. Surprisingly, LDL treatment suppressed organoid clonogenic potential ex vivo compared to control PBS treatment (Fig. 4E, F). Furthermore, LDL treatment altered the morphology of 3D structure derived from crypts (Fig. 4E). PBS-treated crypts grew into organoids characterized by budding structures that harbor stem cells at their base, representing intestinal crypts, interposed by domains carrying all differentiated cell types, representing villi. In contrast, LDL-treated crypts exhibited a higher prevalence of round, fetal-like spheroids (Fig. 4E, G), which have been demonstrated to be mainly composed of proliferating stem cells and lack differentiated cells (19, 27, 28, 29). LDL-treated organoids also had larger surface areas (Fig. 4H). Passaging the primary organoids from LDL-treated group generated significantly more secondary organoids than controls (Fig. 4I). Quantitative RT-PCR analysis showed that several stemness genes were upregulated by LDL treatment, including *Ascl2*, *Axin2*, and *Bmi1* (Fig. 4J). However, there was no change in enterocyte or enteroendocrine cell markers. To further assess if the effect of LDL is dose dependent, we treated organoid cultures with different LDL concentrations. Quantification of organoid growth confirmed a dose-dependent decrease in organoid survival, accompanied by an increase in the percentage of spheroids and secondary organoid formation (Fig. 4K–M). These data indicate that LDL impairs the survival of ISCs but promotes their self-renewal capacity in ex vivo culture.

To further explore if the effect of LDL on ISC function is LDLR dependent, we evaluated organoid-forming ability from both WT and *Ldlr*^−/−^ mice. Interestingly, crypts from both mice demonstrated comparable primary organoid formation rate, spheroid percentage, surface area, and secondary organoid formation rate in the presence of LDL treatment (Fig. 4N–R), suggesting that the effect of LDL treatment on ISC function is independent of LDLR.

### LDL treatment enhances ISC function through the activation of PPAR pathway

**Fig. 5:**
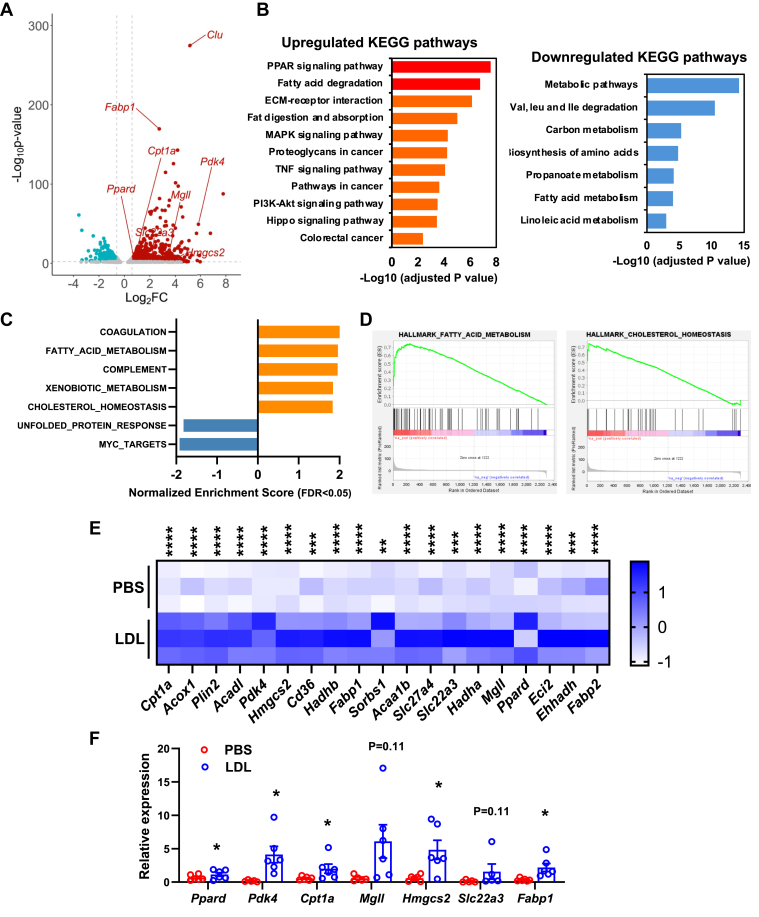
RNA-Seq analysis revealed activation of FAO in LDL-treated organoids. A: Volcano plot of differentially expressed genes in organoids derived from C57BL/6J mice and treated with PBS or LDL (50 μg/ml) for 5 days. B: Kyoto Encyclopedia of Genes and Genomes pathway analysis of the upregulated and downregulated genes in organoids derived from C57BL/6J mice and treated with PBS or LDL (50 μg/ml) for 5 days. C: Gene set enrichment analysis (GSEA) of the top dysregulated pathways in organoids derived from C57BL/6J mice and treated with PBS or LDL (50 μg/ml) for 5 days. D: Enrichment plot of the fatty acid and cholesterol metabolism genes in organoids derived from C57BL/6J mice and treated with PBS or LDL (50 μg/ml) for 5 days. E: Heatmap of selected PPAR targets and genes involved in FAO activated by LDL treatment as in A. F: Real-time RT-PCR analysis of stemness and lineage marker genes in PBS- and LDL-treated organoids. Each dot represents data from one mouse (n = 5–6). Values are means ± SEM. Statistical analysis was performed with paired Student's *t*-test. ∗*P* < 0.05, ∗∗*P* < 0.01, ∗∗∗*P* < 0.001, and ∗∗∗∗*P* < 0.0001.

To unravel the underlying mechanism by which LDL treatment impacts ISC proliferation and self-renewal, we performed RNA-Seq analysis on LDL- and PBS-treated organoids. RNA-Seq analysis revealed 911 genes upregulated and 518 genes downregulated by 1.5-fold in response to LDL treatment (Fig. 5A). Pathway analysis revealed that PPAR signaling and fatty acid degradation were the top enriched pathways among the upregulated genes in LDL-treated organoids (Fig. 5B). Interestingly, the most downregulated pathways were metabolic pathways, particularly those involved in amino acid, carbon, and fatty acid metabolism (Fig. 5B). Gene set enrichment analysis further confirmed that genes involved in fatty acid and cholesterol metabolism were among the top upregulated genes in the LDL-treated organoids (Fig. 5C, D). These included PPAR target genes *Pdk4*, *Cd36*, *Hmgcs2*, and several key genes critical in FAO, such as *Cpt1a* (encodes carnitine palmitoyl-transferase1A, the rate-limiting enzyme of FAO), *Acox1* (encodes acyl-coenzyme A oxidase 1, the first enzyme of FAO), *Acadl* (encodes acyl-CoA dehydrogenase long chain), *Hadha/Hadhb* (encodes hydroxyacyl-CoA dehydrogenase trifunctional multienzyme complex subunit alpha/beta catalyzing the last three steps of FAO), *Eci2* (encodes enoyl-CoA delta isomerase 2 involved in β-oxidation of unsaturated fatty acids), and *Ehhadh* (encodes enoyl-CoA hydratase and 3-hydroxyacyl CoA dehydrogenase) (Fig. 5E). The upregulation of most PPAR targets and FAO genes was validated by real-time RT-PCR analysis (Fig. 5F).

**Fig. 6:**
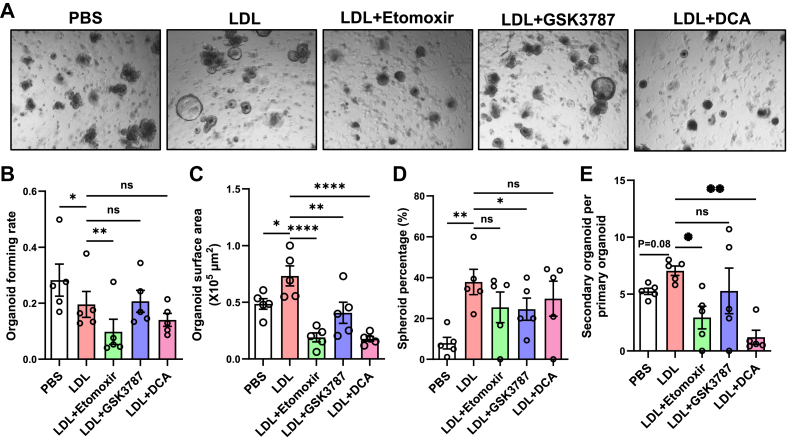
PPAR and FAO mediate the effect of LDL treatment in organoid self-renewal. A: Representative images of WT organoids treated with PBS control, LDL (50 μg/ml), or LDL and etomoxir (100 μM), GSK3787 (2 μM), or DCA (20 mM). B–E: Quantification of organoid-forming rates (B), organoid surface areas (C), the percentage of spheroids (D), and secondary organoid-forming rates (E) from A. Each dot represents the mean of three to four wells from one mouse (n = 5 mice). Values are means ± SEM. Statistical analysis was performed with one-way ANOVA. ∗*P* < 0.05, ∗∗*P* < 0.01, ∗∗∗∗*P* < 0.0001, and ns, not significant.

The PPAR pathway and FAO have been shown to promote ISC proliferation in response to fasting or high-fat diet feeding (19, 20, 30). To further investigate whether the PPAR and FAO pathways contribute to the enhanced ISC function induced by LDL treatment, we pharmacologically suppressed these pathways in LDL-treated organoid cultures using etomoxir (CPT1A inhibitor), GSK3787 (PPARβ/δ inhibitor), and DCA (PDK4 inhibitor). Administration of etomoxir resulted in marked reduction in organoid survival and growth, evidenced by a 50% decrease in organoid-forming rate and a 70% reduction in surface area (Fig. 6A–C). Although etomoxir treatment did not alter organoid morphology (Fig. 6D), it significantly reduced the self-renewal ability revealed, as demonstrated by a ∼60% decrease in secondary organoid-forming rate upon passaging (Fig. 6E). In contrast, GSK3787 inhibited organoid growth without affecting their survival and self-renewal ability, whereas DCA treatment dramatically inhibited both organoid growth and self-renewal capacity (Fig. 6A–E). Taken together, these findings demonstrate that both the PPAR pathway and FAO contribute to the augmented growth and self-renewal ability of organoids following LDL treatment.

## Discussion

The ISCs in the crypt have the challenging task of continuous replenishment of the intestinal epithelium while avoiding uncontrolled proliferation. To maintain this balance, ISC homeostasis is tightly regulated by both intrinsic and extrinsic factors, including niche factors, nutrients, and dietary regimes (31). In this study, we demonstrate that, in addition to dietary lipids, circulating lipids also influence ISC function. Hyperlipidemia, induced by the loss of *ldlr*, an important receptor for plasma LDL clearance, enhances ISC function in vivo. Consistently, LDL treatment promotes stemness and self-renewal capabilities of the crypts, resulting in the formation of fetal-like spheroid structures, increased surface area, and elevated secondary organoid formation ex vivo.

Altered lipid metabolism has long been linked to gastrointestinal cancer (32, 33, 34, 35, 36). Lipidomics analyses have revealed increased levels of specific phospholipids, fatty acids, triglycerides, and cholesterol in gastrointestinal cancer tissues (35, 37). Considering that ISCs are the primary cells of origin for gastrointestinal cancers, it is not surprising that some of these lipids enhance ISC function and contribute to tumorigenesis (19, 20, 22, 30). Although the relationship between serum lipid levels and gastrointestinal cancer remains inconclusive, the majority of studies indicate a positive association between elevated serum triglyceride and cholesterol levels, particularly LDL-cholesterol and free cholesterol levels and an increased incidence of CRC (4, 38, 39, 40). Our data indicate that hyperlipidemia induced by *Ldlr* deficiency enhances ISC function both in vivo and ex vivo, suggesting a potential mechanism by which elevated circulating lipids may promote tumorigenesis. Based on these observations, it is reasonable to consider targeting hyperlipidemia as a potential chemopreventive strategy for CRC. Notably, several clinical studies have reported an association between the use of lipid-lowering drugs, such as statins, and a reduced CRC risk (41, 42, 43, 44). Nevertheless, it would be worthwhile to investigate whether hyperlipidemia could enhance tumorigenesis by crossing *Ldlr*-deficient mice with *Apc* mutant mice or other CRC models.

Interestingly, LDLR has been shown to be overexpressed in the polyps of *Apc*^*min/+*^ intestine, which likely facilitates lipid uptake and subsequent lipid accumulation, thereby supporting the growth of these polyps (45). In a mouse model with *Ldlr* overexpression in the small intestine, it was observed that cell turnover rate is increased as a compensatory response to increased epithelial cell apoptosis (46). In contrast, our data showed that loss of *Ldlr* leads to enhanced ISC proliferation. This effect is likely attributable to hyperlipidemia resulting from *Ldlr* deficiency in the liver, which is supported by our observations that crypts lacking LDLR exhibit growth pattern comparable to those of WT crypts. Indeed, survival analysis of a The Cancer Genome Atlas dataset of CRC patients revealed no difference in overall survival between LDLR high and low expression groups (data not shown), indicating that LDLR expression in the gut per se is unlikely a major determinant of CRC prognosis. Nevertheless, further studies are required to determine whether hyperlipidemia alone is sufficient to drive ISC hyperproliferation in vivo using other hyperlipidemic models to circumvent the potential impact of *Ldlr* deficiency in the intestine, such as *Ldlr* liver-specific knockout mice or hepatic overexpression of PSCK9, which is known to cause hyperlipidemia by facilitating the degradation of LDLR in the liver.

LDL is composed of a variety of lipids, including phospholipids, free cholesterol, cholesteryl ester, and triglycerides. Previous research has established that several of these lipids play a role in modulating ISC function. For instance, dietary factors such as high-fat diet and fasting have been shown to activate PPAR-mediated FAO to boost ISC stemness (19, 20, 30). Additionally, the composition of phospholipids within the intestinal epithelium influences ISC behavior, with increased saturation of membrane phospholipids leading to ISC hyperproliferation (22). Furthermore, elevated cellular cholesterol levels, induced either through enforced cholesterol biosynthesis or high cholesterol diet feeding, can augment ISC function (22). Our study demonstrated that LDL promotes ISC function through the activation of PPAR pathway and its downstream FAO, mirroring the effects observed with high-fat diet feeding or fasting. It is plausible that LDL may increase the delivery of these lipids to ISCs, thereby enhancing ISC function. However, unexpectedly, our ex vivo organoid culture experiments suggest that the effect of LDL on ISC function is likely independent of LDLR. *Ldlr*-deficient crypts respond to LDL treatment similarly to WT crypts. This finding implies that the effect of LDL may involve other lipid components derived from LDL, such as fatty acids, which can be released from triglyceride or cholesteryl ester and taken up by fatty acid transporters like CD36. Moreover, it has been reported that LDL can be internalized via LDLR-independent mechanisms. Although LDL uptake is significantly reduced in *Ldlr*-deficient mice, a residual fraction of circulating LDLs is still cleared by the liver (47). Additionally, Sortlin, a post-Golgi trafficking receptor, has been identified as a mediator of LDL internalization independent of LDLR (48, 49). Other receptors, including Megalin/GP330 and ALK1, have also been shown to facilitate LDL uptake in vitro and/or in vivo (50, 51).

In summary, our studies reveal a significant impact of circulating LDL and its lipid contents on ISC homeostasis. This research underscores a potential mechanistic link between hyperlipidemia and an elevated risk of gastrointestinal cancers.

## Data availability

RNA-Seq data have been deposited at Gene Expression Omnibus (accession number: GSE269253). Raw data generated during the present study are available from the corresponding authors upon request.

## Conflict of interest

The authors declare that they have no conflicts of interest with the contents of this article.
